# Supplementary material for: Tumor Stimulus-Responsive Biodegradable Diblock Copolymer Conjugates as Efficient Anti-Cancer Nanomedicines
Source: J Pers Med. 2022 Apr 27;12(5):698. doi: 10.3390/jpm12050698 (PMC9145326; doi:10.3390/jpm12050698)
Supplement: Supplementary file 1 [file jpm-12-00698-s001.zip › jpm-1686287-supplementary.pdf]

Supporting information

Tumor Stimulus-responsive Biodegradable Diblock Copolymer Conjugates as Efficient anti-cancer nanomedicines

Vladimír Šubr[a], Robert Pola[a],Shanghui Gao[b], Rayhanul Islam[b], Takuma Hirata[b], Daiki Miyake[b], Kousuke Koshino[b], Jian-Rong Zhou[b], Kazumi Yokomizo[b], Jun Fang[b#], Tomáš Etrych[a*]

[a] Institute of Macromolecular Chemistry, Academy of Sciences of the Czech Republic, Heyrovsky Sq. 2, 162 06 Prague 6, Czech Republic,

[b] Faculty of Pharmaceutical Sciences, Sojo University, Ikeda 4-22-1, Kumamoto Nishi-ku, Japan 860-0082.

*Corresponding Author: Tomáš Etrych.

E-mail address: etrych@imc.cas.cz

#Co-coresponding author for the biology part: Jun Fang

E-mail address: fangjun@ph.sojo-u.ac.jp

The authors wish to dedicate the paper to the honor of Hiroshi Maeda, a pioneer in the field of the drug delivery.

[A]

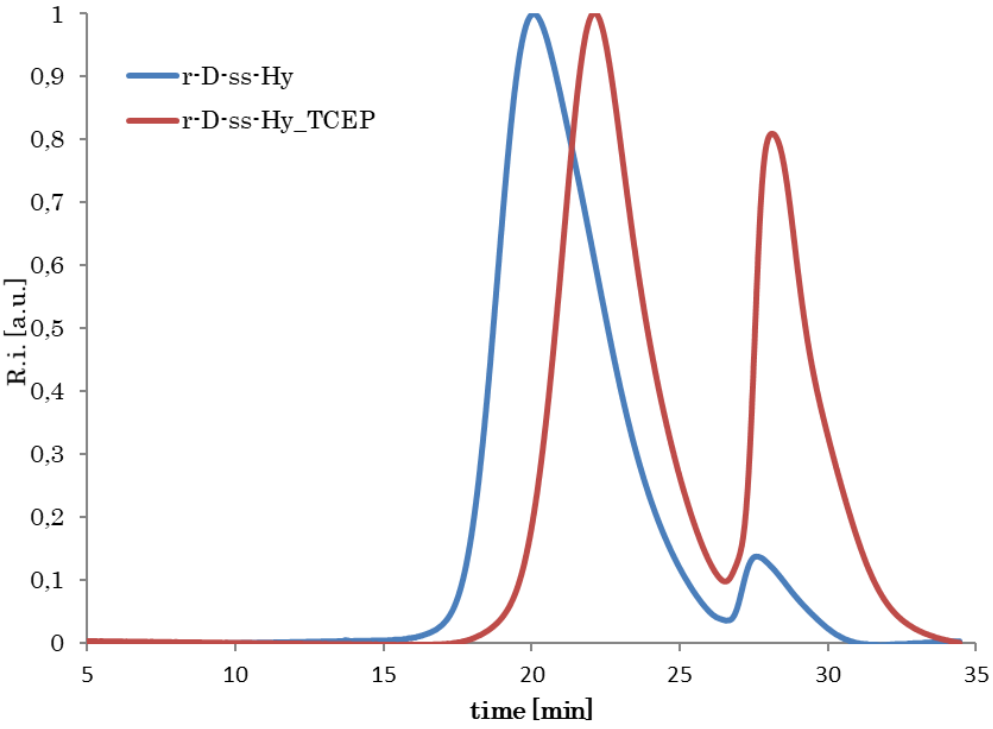
r-D-ss-Hy
r-D-ss-Hy_TCEP
R.i. [a.u.]
time [min]
1
0,9
0,8
0,7
0,6
0,5
0,4
0,3
0,2
0,1
0
5
10
15
20
25
30
35

[B]

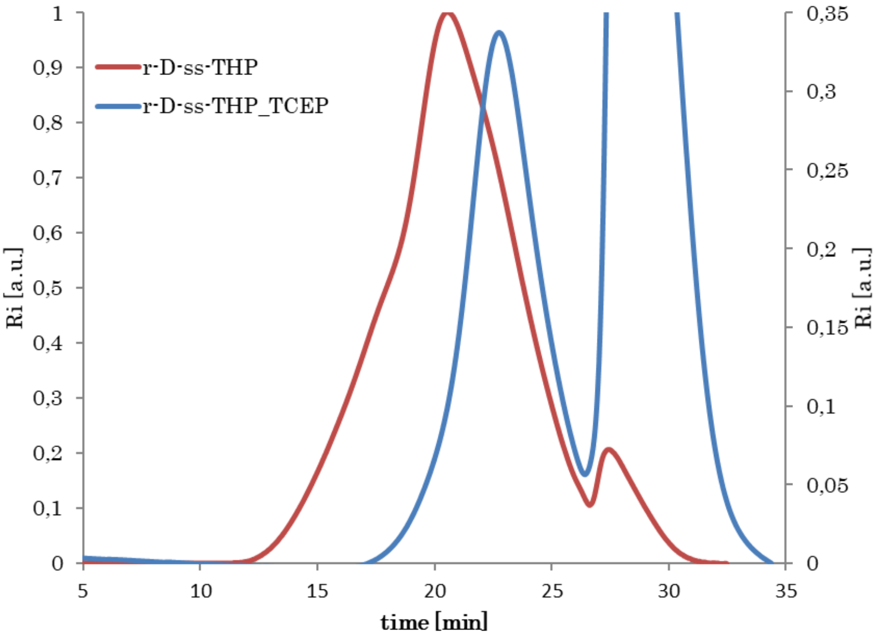
r-D-ss-THP
r-D-ss-THP_TCEP
Ri [a.u.]
Ri [a.u.]
time [min]
1
0,9
0,8
0,7
0,6
0,5
0,4
0,3
0,2
0,1
0
0,35
0,3
0,25
0,2
0,15
0,1
0,05
0
5
10
15
20
25
30
35

[C]

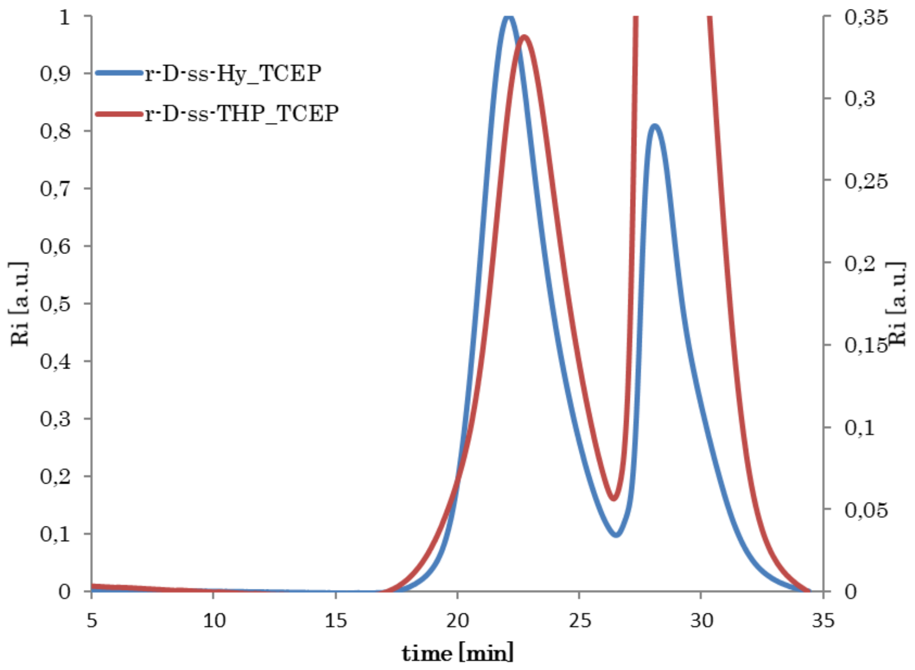

SI_Figure S1. GPC profiles of diblock polymers before and after the reduction: Comparison of GPC profiles of **r-D-ss-Hy** before and after incubation with TCEP [A], Comparison of GPC profiles of **r-D-ss-THP** before and after incubation with TCEP [B], Comparison of GPC profiles of **r-D-ss-Hy** and **r-D-SS-THP** after incubation with TCEP [C].
